# Usual hospital care versus post-abortion care for women with unsafe abortion: a case control study from Sri Lanka

**DOI:** 10.1186/1472-6963-14-470

**Published:** 2014-10-31

**Authors:** Carukshi Arambepola, Lalini C Rajapaksa, Chandani Galwaduge

**Affiliations:** Department of Community Medicine, Faculty of Medicine, University of Colombo, Kynsey Road, Colombo 8, Sri Lanka; Former Professor in Community Medicine Department of Community Medicine, Faculty of Medicine, University of Colombo, Kynsey Road, Colombo 8, Sri Lanka; UNFPA, Bauddhaloka Mawatha, Colombo 7, Sri Lanka

**Keywords:** Post-abortion-care, Contraception, Discrimination, Comparison groups

## Abstract

**Background:**

Good quality post-abortion-care (PAC) is essential to prevent death and long-term complications following unsafe abortion, especially in countries with restrictive abortion laws. We assessed the PAC given to women following an unsafe abortion, compared to the routine hospital care following spontaneous abortion or unintended pregnancy carried to term in Sri Lanka.

**Methods:**

A case–control study was conducted in Sri Lanka among 171 cases following unsafe abortion, 638 controls following spontaneous abortion (SA-controls) and 600 women following delivery of an unintended pregnancy (TUP-controls) admitted to same hospitals during the same period. Care provided was assessed using interviewer-administered-questionnaires and in-depth-interviews at hospital discharge and in a sub-sample, at 6–8 weeks post-discharge. Differences in care were assessed using chi-square tests.

**Results:**

Mean age of cases was 30.6 years (SD = 6.6); 21.1% were primis. 60.8% cases developed sepsis and 12.3% organ failure. Cases received timely, complete and safe emergency treatment with no difference to SA-controls (p > 0.05): removal of retained products of conception medically (14.6% cases versus 19.4% SA-controls) or surgically (73.7% versus 75.1%), within 24 hours of admission (63.5% versus 52.8%), under anaesthesia (84.1% versus 92.3%) and intravenous antibiotics (91.2% versus 31.0%). Despite this equitable treatment, cases were dissatisfied with their overall care during hospital stay, predominantly due to verbal harassment of health-care-providers on their abortion status (57.9% versus 19.3% SA-controls, p < 0.05). Ward doctors provided the best care to cases in all aspects, except compared to SA-controls in explaining women’s health status (60.2% versus 77.7%), and compared to TUP-controls in providing information on contraceptive methods (14% versus 24.3%), service availability (13.5% versus 24.7%) and assistance in decision-making on contraception (13.5% versus 21.3%). Ward-midwives contributed none to family-planning care of cases. At 6–8 weeks, 48.9% of cases were on contraceptive methods, predominantly short-term, compared to 85.3% of TUP-controls, predominantly long-term methods (p < 0.01).

**Conclusions:**

Despite equitable emergency treatment, care following unsafe abortion was deficient in post-abortion counselling, education and family planning services. Engagement of public-health staff for follow-up care was inadequate. Perceived dissatisfaction of overall care was owing to discrimination related to their abortion status.

## Background

Unmet need for contraceptive services results in a large number of unwanted pregnancies. Although developed and developing regions show similar rates of induced abortion amongst women with such pregnancies, unsafe abortions are highly concentrated in the developing countries [1]. Especially in countries where abortion is legally restricted except to save a woman’s life, safe abortion services are out-of-reach for many facing unwanted pregnancies [2].

Unsafe abortion is ‘any procedure used for terminating an unwanted pregnancy either by persons lacking the necessary skills or in an environment lacking the minimum medical standards, or both’ [3]. Apart from death, it is well-known to cause sepsis, retained products of conception, haemorrhage, organ damage and long-term consequences such as pelvic inflammatory disease, tubal occlusion and secondary infertility [1]. Studies have shown that one in 5 women suffers a reproductive tract infection following an unsafe abortion [4]. These consequences are crucial in Asia, where unsafe abortion is a problem predominantly among the young and the poor, with a higher tendency towards life-threatening complications [5–7]. It emphasises the need for good quality post-abortion care in this region to save life and to minimise long-term consequences of unsafe abortions.

The World Health Organization (WHO) estimates that 10-50% of women who undergo unsafe abortions require medical care [8]. The 1994 International Conference on Population and Development (ICPD) in Cairo, in its consensus Programme of Action, recognised unsafe abortion as a major public health concern and called for all women to have access to post-abortion care (PAC), regardless of the legal status of abortion [9]. PAC is a comprehensive strategy that includes both medical and preventive services on emergency treatment of complications caused by unsafe procedures; provision of post-abortion counselling, education and family planning services; and engagement of the reproductive healthcare system in the care [10]. In countries where abortion is legally restricted, PAC ensures that women receive care that is complete, appropriate and prompt (CAP) [11].

Millennium Development Goal 5 is to improve maternal health by reducing maternal mortality by 75% from 1990 to 2015 and providing universal access to reproductive health [12]. Sri Lanka, a country in South Asia is fully-geared to achieve this goal through its national safe motherhood programme, which has successfully reduced the maternal mortality from 2680 per 100,000 live births in 1936 to 33 by 2010 [13]. Further reduction requires specifically targeting the most easily preventable causes of death, such as sepsis following unsafe abortion that assumes the fourth place in maternal mortality in Sri Lanka [14]. Although state-owned health facilities do not provide abortion services owing to the country’s legally restrictive abortion policy, these hospitals are liberally accessed by women who develop complications following an unsafe abortion. It is therefore essential that all institutions are well-equipped to provide PAC for safeguarding women’s health.

Health status of Sri Lanka is sustained by the Government policy of ‘free health for all’, which gives its people access to state-owned health facilities within any part of the country [15]. Through this healthcare system, specialist obstetric services are offered at all district-level hospitals while public health services including domiciliary care are provided throughout the country by public-health-midwives (PHM) as the grass-root-level workers. Despite such comprehensive services [13], it is not well-documented whether PAC is implemented in its optimal capacity, given the highly restrictive status of abortion, and strong religious and socio-cultural stigma attached to it in Sri Lanka. Furthermore, whether women seeking post-abortion care would face discrimination because of their abortion status has not been systematically studied. Although several studies have been conducted on hospital care received by women following an induced abortion in legally-restrictive settings [16–20], differentials of care in comparison to the routine care received by women who had a planned pregnancy but ended up in a similar emergency condition such as a spontaneous abortion, or women who have had a similar unintended pregnancy but carried to term, have not been assessed. This study was conducted to assess the post-abortion care following an unsafe abortion in comparison to the routine care in hospitals in Sri Lanka.

## Methods

An unmatched case–control study was conducted in nine hospitals in eight out of the 24 districts in Sri Lanka. Five of these hospitals were selected based on the highest frequency of all types of abortions reported in Indoor Morbidity and Mortality Registers for each district [Medical Statistics Unit, unpublished]. Two hospitals were intentionally selected to ensure representation of the Muslim and estate sector Tamil populations. In the district of Colombo, both largest apex referral tertiary hospitals in the country were included.

### Study population

The study recruited women admitted to the selected hospitals for care following an unsafe abortion as ‘cases’ and for comparison, the following two groups of women admitted to same hospitals during the same study period as ‘controls’:

- Control group 1: Women admitted for care following a spontaneous abortion following a planned pregnancy (SA-controls)
- Control group 2: Term unintended pregnancy (TUP-controls) – Women admitted for delivery of an unintended pregnancy carried to term.

**Figure 1:**
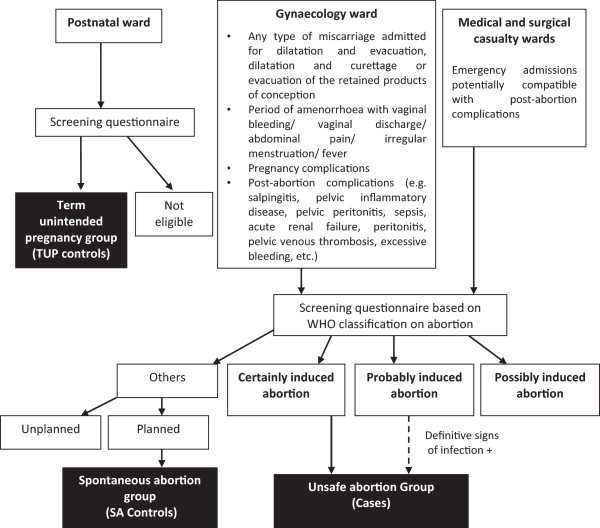
**Flow diagram used to categorise women into ‘Cases’, ‘SA-controls’ (spontaneous abortion group) and ‘TUP-controls’ (term unintended pregnancy group).**

During recruitment, all women admitted to the gynaecology and medical/surgical casualty wards were screened consecutively for signs and symptoms suggestive of an ‘abortion’ (Figure 1). Of them, the women with a confirmed diagnosis of ‘induced abortion’ were identified based on the World Health Organization (WHO) criteria [21] under three categories: ‘certainly induced’ based on woman’s statement and/or genital trauma or evidence of manipulation or foreign body in the genital tract (n = 122); ‘probably induced’ based on sepsis/peritonitis *and* unintended pregnancy (n = 161); and ‘possibly induced’ based on sepsis/peritonitis *or* unintended pregnancy (n = 539). An ‘unintended pregnancy’ was defined by the pregnancy of a woman contracepting during the cycle of conception or not contracepting due to reasons other than desired pregnancy [21]. From these women, ‘cases’ were identified for the study by excluding all women in the ‘possibly induced’ abortion group (n = 539) and those in the ‘probably induced’ abortion group whose signs of infection seemed less-definitive (n = 112). This ensured that cases represented only unsafe abortions and did not include women who probably had a spontaneous abortion but were misclassified into the ‘probably’ or ‘possibly’ induced abortion groups because of the history of an unintended pregnancy, which was one criterion used to identify an induced abortion.

Out of all women who were excluded by the initial screening for induced abortion, women to be included in the SA-control group were identified by a confirmed diagnosis of ‘spontaneous abortion’, as defined by any abortion that did not conform to the criteria of an induced abortion plus a confirmed history of a planned pregnancy [21] (Figure 1).

The women in TUP-control group were identified in postnatal wards using a systematic sampling method, by confirming the delivery of a term unintended pregnancy [21]. The minimum sample size required for the study was 159 cases and 600 in each control group, based on 80% power to detect potential associations between cases and controls at 5% alpha error; 20% minimum probability of exposure in the controls; odds ratio (OR) of 2; and 1:4 unmatched case–control ratio.

### Study instruments and data collection

The study was approved by the Ethics Review Committee of the Faculty of Medicine, University of Colombo, Sri Lanka. Official permission was obtained from the Provincial Directors of Health Services and Directors of the selected hospitals. After obtaining informed verbal consent, data were collected from all participants at an exit-interview using an interviewer-administered-questionnaire. The interviewers were Pre-intern medical officers who were not involved in providing care. They were trained by a group of Psychologists and experts in qualitative research in recruiting and obtaining sensitive information from women. The questionnaire developed by the WHO for multi-centre hospital-based descriptive studies on abortions [21] was culturally modified for data collection. Sections in the questionnaire on socio-economic characteristics, care provided by different ward staff categories at the time of emergency treatment (as perceived by women themselves) and the overall satisfaction of hospital care were administered to cases and the SA-control group. ‘Emergency treatment’ was defined by any medical treatment given or surgical procedure carried out for treating the complications of abortion. Hospital patient records were perused for verifying their post-abortion complications, treatment administered and clinical outcomes. Sections in the questionnaire on contraceptive education and family planning services in the hospital provided by different ward staff categories (as perceived by women themselves) were administered to cases and the TUP-control group.

At 6–8 weeks following discharge, investigators conducted telephone interviews in a sub-sample of cases and TUP-controls (N = 95 each) to assess the family planning services received since discharge. In addition, the principal investigator conducted in-depth interviews with a group of consultant gynaecologists (N = 6), ward nurses (N = 8) and midwives (N = 8) to explore their views on post-abortion care. This sample size was decided based on saturation method.

### Data analysis

Statistical Package for the Social Sciences (SPSS) Version 20.0 was used for data analysis. Post-abortion complications, treatment and clinical outcomes, care received at the time of emergency treatment and the overall satisfaction of hospital care of cases were compared with that received by women in the SA-control group, whilst family planning education and services received during and 6–8 weeks after their hospital stay were compared with that received by women in the TUP-control group. Significance of the differences was assessed using chi-square test.

## Results

The study consisted of 171 cases, 638 women in the SA-control group and 600 in the TUP-control group. The mean age of cases was 30.6 years (SD = 6.6) compared to 28.5 (SD = 5.7) of the SA-controls and 30.5 (SD = 6.3) of the TUP-controls. Cases consisted of 21.1% primis (SA-controls = 51.9%; TUP-controls = 13.8%), 81.9% married (SA-controls = 100%; TUP-controls = 98.3%), 32.4% educated beyond upper secondary school (SA-controls = 54.6%; TUP-controls = 44.5%) and 40.4% employed (SA-controls = 24.5%; TUP-controls = 24.8%).

### Treatment received for post-abortion complications

On admission, all cases except 67 women with certainly induced abortion (104; 60.8%) showed definitive clinical signs of infection (septicaemia = 70; peritonitis = 2; endometritis = 16; tetanus = 1; high-grade fever = 15) compared to only two in the SA-control group. Compared to one in the SA-control group, 21 (12.3%) cases went into organ failure (hypovolaemic shock = 14; cardiac failure = 2; coagulation defects = 3; renal failure = 1; anaemia = 1). Two cases (1.25%) died following complications.

Table 1 compares the emergency treatment received by the cases and SA-controls. The retained products of conception were removed in cases either surgically (73.7% cases versus 72.7% SA-controls) or medically (14.6% versus 19.4%). Surgical procedures of cases included manual vacuum aspiration (MVA) (57%), dilation and evacuation (D & E) (31%), manual removal of products (8%) and abdominal hysterectomy (4%). The majority of cases underwent these procedures within 24 hours of admission (63.5% versus 52.8%) and under anaesthesia (84.1% versus 92.3%). Almost all cases (91.2% versus 31.0%) were treated with intravenous antibiotics such as Cephalosporins and Metronidazole. 67.3% of cases received intravenous fluids (1.9 litres on average per person) while 41.4% were infused blood/blood products (0.9 litres on average per person). There was no significant difference between cases and SA-controls in relation to any delay in initiating treatment, choice for evacuation of retained products and administering pain relief (p > 0.05).

**Table 1:** **Comparison of the emergency treatment received by women with unsafe abortions (Cases = 171) and women with spontaneous abortion (SA-controls = 638)**

Emergency treatment		Cases		SA-controls	Significance
	No.	%	No.	%	
***Removal of retained products:***					
Time since admission to treatment*					
Too long	28	16.4%	72	11.3%	ϰ^2^ = 3.2; df = 1
Appropriate	143	83.6%	566	88.7%	p = 0.07
**Procedure used**					
Medical induction	25	14.6%	124	19.4%	-
Surgical removal	126	73.7%	464	72.7%	
Both surgical & medical removal	0	0.0%	15	2.4%	
None	20	11.7%	35	5.5%	
Surgical removal within 24 hours**	80	63.5%	253	52.8%	ϰ^2^ = 4.6; df = 1
					**p = 0.03**
Anaesthesia for surgical removal**	106	84.1%	442	92.3%	ϰ^2^ = 7.76; df = 1
					**p = 0.005**
***Supportive care:***					
**Intravenous antibiotics given**	156	91.2%	198	31.0%	ϰ^2^ = 198; df = 1
					**p = 0.00**
**Pain relief given**	159	93.0%	585	91.7%	ϰ^2^ = 0.3; df = 1
					p = 0.5
***Intravenous fluids/products given:***					
**Blood**	54	31.6%	4	0.6%	
**FFP/cryo/platelets**	23	13.5%	0	0.0%	
**Normal saline**	106	62.0%	158	24.8%	
**Hartman**	34	19.9%	18	2.8%	
**5% dextrose**	18	31.6%	20	3.1%	

*Length of time, as perceived by the patients.

**Includes 126 unsafe abortions and 479 spontaneous abortions who underwent a surgical procedure.

p value in bold print, if significant at 0.05 level.

Table 2 compares the women’s perception of care provided by different ward staff categories to the cases and SA-controls at the time of emergency treatment. Of all categories, doctors provided the best care to cases, prior to and during their treatment. The majority of cases received an explanation from doctors about their surgical/medical procedure (64.9% versus 56.7%) and health status (60.2% versus 77.7%). However, the explanation on health status was significantly less among the cases compared to SA-controls (p < 0.01). Both groups were not given enough opportunities to clarify doubts on their health status or procedure (<30%).

**Table 2:** **Comparison of the care provided by different staff categories at the time of emergency treatment to women with unsafe abortion (Cases = 171) and women with spontaneous abortion (SA-controls = 638)**

Care related to emergency treatment	Ward staff category (%)*					
		Ward doctor			Ward nurse	
	Cases	SA	***p value***	Cases	SA	***p value***
**Prior to medical/surgical procedure**						
• Spoke before initiating treatment	82.5	87.3	*0.1*	38.6	26.3	***0.002***
• Explained the current health status	60.2	77.7	***0.001***	23.5	19.6	*0.3*
• Opportunities given to clarify doubts	25.7	25.2	*0.9*	11.7	13.6	*0.5*
**During and around the time of procedure**						
• Explained the procedure	64.9	56.7	*0.05*	24.6	28.8	*0.3*
• Helped in understanding the procedure	46.8	45.3	*0.7*	20.5	25.7	*0.2*
• Opportunities given to clarify doubts	23.4	28.2	*0.2*	16.4	21.9	*0.1*

*All percentages were calculated out of 171 in the unsafe abortion (cases) group and 638 in the spontaneous abortion (SA-controls) group.

p value in bold print, if significant at 0.05 level.

### Post-abortion counselling, education and family planning services

Neither of the two groups of women with abortions formally received any counselling services in the ward setting. Depending on the availability, a few hospitals referred patients for counselling to out-patient clinics.

**Figure 2:**
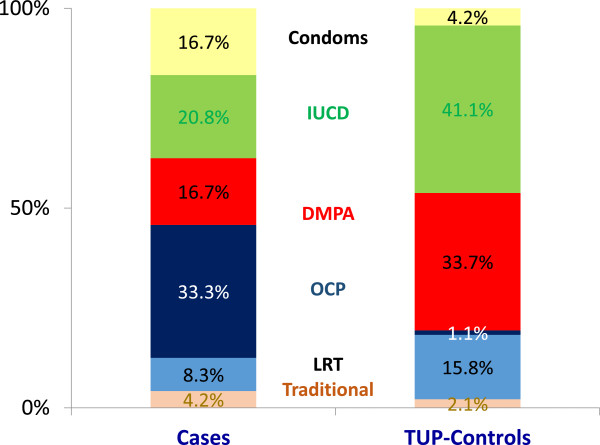
**Methods of contraception use at 6–8 weeks after hospital discharge among women with unsafe abortion (Cases = 171) and postpartum women following an unintended term delivery (TUP-controls = 600).** IUCD=Intra uterine contraceptive device; DMPA=Depot medroxyprogesterone acetate; OCP=Oral contraceptive pills; LRT=Ligation & resection of tubes.

As shown in Table 3, doctors provided the best care to cases, by seeing more at discharge than the other ward staff categories. However, this care with regards to education on contraceptive methods (14.0% versus 24.3%) and family planning services (13.5% versus 24.7%), opportunities for clarifying doubts (12.3% versus 22.8%) and deciding on a suitable contraceptive method (13.5% versus 21.3%) was significantly less among the cases compared to that given to TUP-controls. Ward-midwives saw women only if they were postpartum mothers (24.7%) and on in-depth interview, they claimed that the provision of family planning information was not within the purview of their duties, but the responsibility of the PHM. In-depth interviews with the Consultants revealed that poor family planning education was due to lack of time. While some Consultants believed that this responsibility should lie with the public-health staff, some agreed that the best opportunity for initiation of family planning education would be before hospital discharge. With regards to advocating contraception, their practices varied: a few cases underwent sterilization methods before hospital discharge; some were followed-up at 6–8 weeks; while the majority were lost to follow-up.Of the 95 cases followed-up, only 49 provided information at 6–8 weeks. Of them, only 48.9% (24 out of 49) were practising a method of contraception, leaving the majority in this group vulnerable to another unintended pregnancy. In comparison, 85.3% (81 out of 95) of TUP-controls were on contraception. The cases favoured modern short-term contraceptive methods whilst TUP-controls favoured permanent or long-term temporary methods (Figure 2).

**Table 3:** **Comparison of the care on contraception provided by different staff categories to women with unsafe abortion (Cases = 171) and postpartum women following an unintended term delivery (TUP-controls = 600)**

Care related to contraception	Ward staff category (%) ^#^					
		Doctor		Nurse		Midwife
	Cases	TUP	Cases	TUP	Cases	TUP
**Seen at discharge**	59.1	50.0^*^	17.5	21.7	0.6	24.7^**^
**Provided information on:**						
• Risk of another unwanted pregnancy	27.5	12.8^**^	8.8	2.5^**^	0.6	1.8
• Use of contraception to prevent future unwanted pregnancies	22.2	27.3	7.0	11.0	0.0	9.0
• Currently available methods	14.0	24.3^**^	3.5	10.3^**^	0.0	8.7^**^
• Places to purchase/obtain these methods	13.5	24.7^**^	2.3	13.3^**^	0.0	8.5^**^
**Provided opportunities to clarify doubts**	12.3	22.8^**^	2.3	7.5^*^	0.0	8.0^**^
**Was helpful in deciding future contraception**	13.5	21.3^*^	2.9	6.8	0.0	7.8^**^

^#^All percentages were calculated out of 171 in the unsafe abortion (cases) group and 600 in the unintended term pregnancy (TUP-controls) group.

*p < 0.05.

**p < 0.001.

### Overall satisfaction of the hospital care

The majority of cases were satisfied with the care provided at the time of emergency treatment (55.6%) but were significantly less, compared to the SA-controls (76.8%) (p < 0.05). As for the satisfaction of the overall care provided during their hospital stay, only 42.1% cases were satisfied compared to 80.7% SA-controls (p < 0.01). The dissatisfaction was mostly due to verbal harassment related to disclosure of their abortion status (Table 4). This harassment was mainly carried out by the minor staff and in some instances, to the extent of breaching confidentiality of the information probed into, by revealing the abortion status to family members, staff and other patients.

**Table 4:** **Comparison of the satisfaction of the overall care provided by ward staff during the hospital stay (as perceived by women themselves) to women with unsafe abortion (Cases = 171) and women with spontaneous abortion (SA-controls = 638)**

Overall satisfaction of the care received during hospital stay		Cases		SA-controls
	No.	%	No.	%
Treated well throughout	72	42.1%	515	80.7%
Treated well but was harassed for not revealing facts about the abortion	66	38.6%	2	0.3%
Treated well only after revealing facts about the abortion	15	8.8%	-	-
Treated well initially but was harassed after revealing facts about the abortion	10	5.8%	-	-
Treated well but poorly informed of the condition	4	2.3%	62	9.7%
Confidentiality was breached	7	4.1%	-	-

## Discussion

This is the first study in South Asia highlighting the differentials in the post-abortion hospital care received by women following an unsafe abortion (cases), compared to the routine care received by two comparable groups of women (SA-controls and TUP-controls) in a setting with restrictive abortion laws. Cases received emergency treatment that was timely, complete and safe, with no difference to SA-controls. Despite this equitable care, cases were less satisfied with their care owing to harassment by ward staff related to the stigma associated with their abortion status. Ward doctors provided the best care throughout their hospital stay, but were less competent than for SA-controls in explaining their health status, providing information on family planning services and helping with the decision-making on future contraception. It was remarkable that the ward-midwife provided care only to postpartum mothers, and that all ward staff categories provided fewer opportunities for cases to clarify their doubts related to care. Compared to TUP-controls, contraceptive practices at 6–8 weeks of hospital discharge was poorer among the cases. Cases favoured modern short-term contraceptive methods while TUP-controls favoured modern permanent or long-term methods.

Since obtaining a diagnosis of unsafe abortion is crucial in settings where reporting is deterred by legal, ethical and moral concerns, our study used a robust methodology to distinguish unsafe abortions from spontaneous abortions (Figure 1). Obtaining information at exit interviews by pre-intern medical doctors specifically trained for this purpose and not involved in the patients’ management ensured the validity of data. However, this study is limited by not applying a validated tool to assess satisfaction of care. Bjertnaes et al. [22] reported that the most important predictors for overall patient satisfaction within hospitals are patient-reported experiences and fulfilment of expectations. We assessed only a few of these aspects.

Emergency abortion care includes prompt and appropriate treatment that should be available at every district-level hospital, with established protocols for service delivery and comprehensive training for assuring the quality of care [23]. In developing countries, this care is often provided in a crisis situation [16–19]. Delays before treatment as long as 5–8 (14.5%) and 9–12 (7.3%) hours have been reported [16]. Although no emergency procedure should be performed without pain relief, it is practised widely as a punitive measure for women who undergo induced abortions [24]. In contrast, our study demonstrates that the emergency treatment received by women following unsafe abortion was not different from the usual hospital care, in relation to delay in initiating treatment, evacuation procedure used, pain relief and supportive care.

As in other studies [25–27], sepsis and haemorrhage were the commonest complications of unsafe abortion. However, unlike in many of these studies, complications led to fewer deaths. This may be either due to complications becoming less severe with more fee-levying institutions providing illegal yet safe abortion services or due to skilled care given by PAC-providers. In Sri Lanka, technical skills of doctors are maintained at an optimal level through formal training of specialists and in-service training of non-specialists. Furthermore, every maternal death in the country is investigated during district and national maternal mortality reviews and thereby, the services being constantly reviewed. Research indicates that such internal audits supplemented by external confidential enquiry strengthen the quality assurance of emergency abortion care [28].

Sixteen systematic reviews provide evidence for fewer complications associated with the removal of retained products medically after 14 weeks of gestation; surgically at early gestational ages; and with prophylactic antibiotics [29]. Furthermore, MVA under local anaesthesia is considered safer, faster and more effective with shorter hospital stay than sharp curettage under general anaesthesia [16, 18, 30, 31], which is still commonly practised in developing countries. In our study, none underwent sharp curettage. Although sublingual misoprostol is accepted with high levels of satisfaction and side effect tolerability among females [16, 29, 32], its use was limited in PAC as it is not yet a registered drug in Sri Lanka.

A notable finding of our study was the dissatisfaction of care provided to the majority of cases, despite receiving equitable emergency treatment. Consistent with previous studies [33, 34], this dissatisfaction was influenced by the harassment faced during disclosure of their abortion status. Harassment was mainly by the minor staff and in some instances, to the extent of breaching confidentiality of the information probed into. In PAC, it is the duty of health managers to protect the clients’ information against unauthorised disclosure by creating a respectful environment, with physical space for assuring privacy [35], a wide-range of skills for building rapport with women in a culturally-attuned empathic manner and attitudinal changes at all levels of PAC providers to treat them with dignity, so that women are comfortable in sharing their abortion history with care providers [17, 34, 36]. Sri Lanka Government health policy aims to facilitate equity through increased access to health services and quality of care [15]. The deficiencies identified in this study should be taken into consideration in reaching these targets.

Our study highlights areas for improvement in care, with regards to psychological counselling and opportunities given to women for clarifying doubts. Despite several studies highlighting the importance of addressing emotional sensitivities surrounding the event [35, 37–39], providing such personalised care in free healthcare settings is often challenged by patient over-crowding that limits the time spent per patient. In the case of abortion, most of this time is used for eliciting a history as sensitive as abortion. A study in Finland highlighted similar deficiencies in the communication part of care, with 30% preferring a discussion with a physician or nurse after abortion on psychological effects of abortion [40]. This study recommended training mid-level providers and a supervisory system that holds the health staff accountable for conducting high quality information and counselling sessions.

The key recommendations in PAC include ‘contraceptive methods, accurate information, sensitive counselling and referral for on-going services to all women who have experienced abortion’ [10]. Compared to the postpartum period, ovulation occurs as early as day 11 following a first trimester pregnancy loss. Thus, the best practice is to provide preferably long-term contraception prior to hospital discharge. However, many hospital-based studies [41–44] including ours, show that timely initiation of family planning following unsafe abortions was less than 50%. In Zambia, of the 78% of women treated for abortion complications who were willing to receive information on family planning, only 33% received it, while none was offered a method to take home despite 44% indicating their willingness for initiation [8]. Such limited information and missed opportunities provided in hospitals for abortion cases on contraception was evident in our study and elsewhere [38–40]. In addition, our study showed poor counselling by hospital staff, with more post-abortion cases opting for temporary methods, compared to long-term methods among the postpartum women. Furthermore, the hospital staff showed divided opinion on the responsibility of promoting contraception to cases. All this is crucial in Sri Lanka, since women following induced abortions would be reluctant to access field family planning services, in fear of legal and moral implications. This highlights the need for bridging the gap between field and hospital PAC providers by working closely through a formal notification system that refers women directly to the public health services. Furthermore, our study identifies the ward midwife as a valuable resource that could be utilised more efficiently, by incorporating family planning counselling for post-abortion women as an integral part of her prime duties. This recommendation is supported by a review conducted in 19 countries [5].

## Conclusions

Despite equitable emergency treatment of post-abortion complications, deficiencies were noted in care with regards to provision of post-abortion counselling, education and family planning services among women seeking hospital care following unsafe abortion. Their dissatisfaction on the overall care during hospital stay was largely related to discrimination by care-providers based on their abortion status. Integration with the public health network was inadequate with no mechanism for follow-up care. Utilising the ward midwife in family planning, establishing non-threatening physical environments within wards and improving positive attitudes of all PAC-providers are recommended. Our study findings would be further important, if Sri Lanka is to adapt legislation on broad grounds for abortion as a pragmatic public health approach for further reduction in maternal mortality. With the deficiencies in care and intensity of discrimination highlighted in this study, it is unlikely that women would freely access the abortion services in state hospitals nor would the providers be ready to provide these services.
